# DISPARITIES IN LONELINESS AMONG OLDER ADULTS IN THE UK: AN EVIDENCE OVERVIEW AND CRITICAL EVALUATION

**DOI:** 10.1093/geroni/igad104.1394

**Published:** 2023-12-21

**Authors:** Christina Victor

**Affiliations:** Brunel University-London, Uxbridge, England, United Kingdom

## Abstract

We propose that there are three elements to healthy ageing: physical, mental and social. As a concept social health is less well researched although there is a significant literature on specific aspects of this concept such as loneliness or isolation. Researchers in the USA and Europe focussing on inequality in later life health outcomes have predominantly emphasised inequalities in physical and/or mental health. It is only comparatively recently that attention has been given to addressing disparities in aspects of social health because of the wide-spread stereotype that ‘all older adults’ are lonely and isolated. UK studies consistently demonstrate that specific individual characteristics such as gender, marital status, household composition, physical/mental health, ethnicity and socio-economic status are associated with higher risk of loneliness. Interventions for loneliness are rarely effective. We critique this evidence base on three grounds. First analysis of loneliness disparities focuses on individual characteristics rather than considering an intersectional approach that addresses the complexity and multiplicity of characteristics and identities that each of us embody and how these are associated with loneliness and other social health outcomes. Second research evidence is predominantly cross-sectional with some longitudinal studies. We argue that to fully understand loneliness and other late life social health disparities we need to adopt a lifecourse approach. Thirdly analysis of disparities in loneliness emphasises individual characteristics rather than adopting an ecological perspective which incorporates these, the effects of time as well as macro-level factors such as cultural norms and meso neighbourhood type, urban design and community cohesion.

